# The Multiple Roles of FGF Signaling in the Developing Spinal Cord

**DOI:** 10.3389/fcell.2017.00058

**Published:** 2017-06-02

**Authors:** Ruth Diez del Corral, Aixa V. Morales

**Affiliations:** ^1^Department of Cellular, Molecular and Developmental Neurobiology, Cajal Institute, Consejo Superior de Investigaciones CientíficasMadrid, Spain; ^2^Champalimaud Research, Champalimaud Centre for the UnknownLisbon, Portugal

**Keywords:** spinal cord, spinal cord injury, neuromesodermal progenitors, neural stem cells, patterning, neurogenesis, caudal extension, FGF

## Abstract

During vertebrate embryonic development, the spinal cord is formed by the neural derivatives of a neuromesodermal population that is specified at early stages of development and which develops in concert with the caudal regression of the primitive streak. Several processes related to spinal cord specification and maturation are coupled to this caudal extension including neurogenesis, ventral patterning and neural crest specification and all of them seem to be crucially regulated by Fibroblast Growth Factor (FGF) signaling, which is prominently active in the neuromesodermal region and transiently in its derivatives. Here we review the role of FGF signaling in those processes, trying to separate its different functions and highlighting the interactions with other signaling pathways. Finally, these early functions of FGF signaling in spinal cord development may underlay partly its ability to promote regeneration in the lesioned spinal cord as well as its action promoting specific fates in neural stem cell cultures that may be used for therapeutical purposes.

## Introduction

The spinal cord is the most caudal part of the nervous system which is responsible for body motion, including locomotion, somatosensation and the control of basic functions of the autonomous nervous system. During development, in addition to the neurons that reside within the spinal cord, it provides neural crest cells for the formation of sensory ganglia, ganglia of the autonomous system and for the enteric nervous system. A fundamental aspect of spinal cord development is its relation to the organs and muscles it innervates. Thus, spinal cord development appears highly coordinated in space and time with the caudal extension that accompanies the development of the whole body.

**Figure 1:**
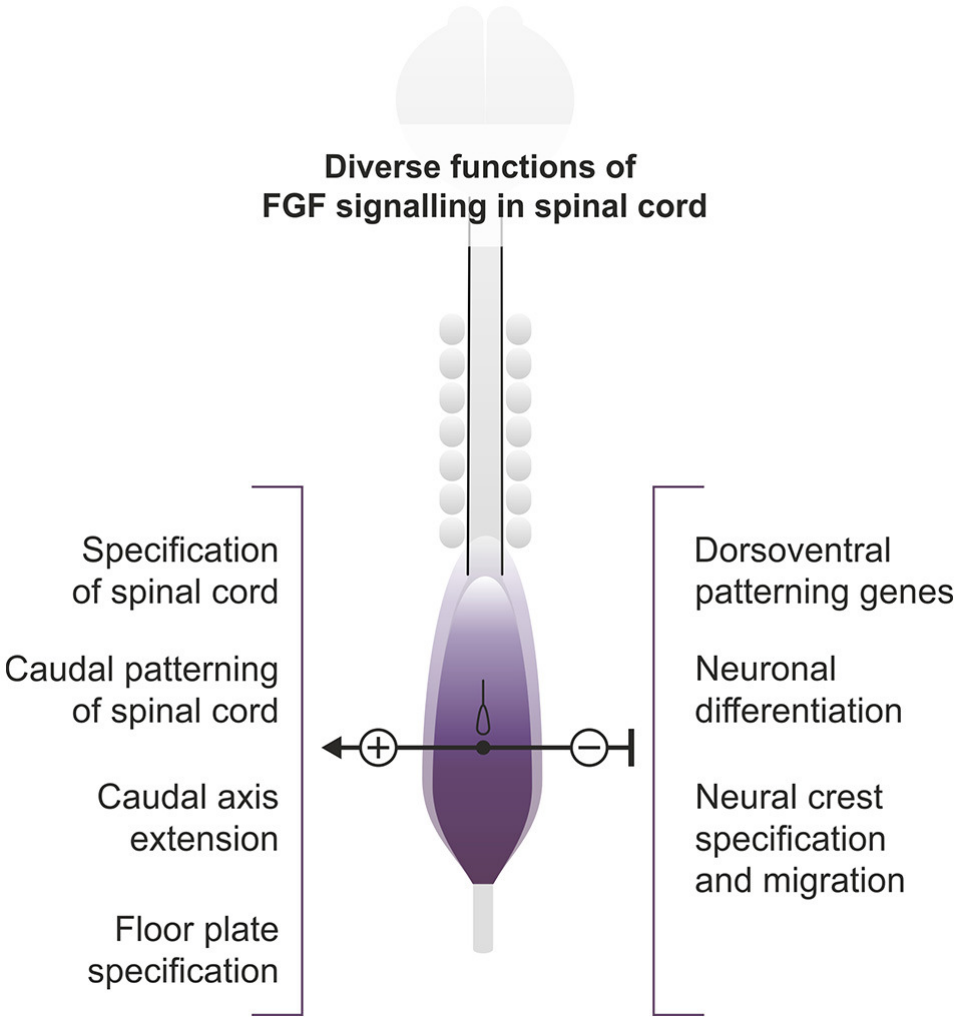
**Roles of FGF signaling in the extending spinal cord**. Diagram representing a 7-somite stage chick embryo and showing the region of active caudal FGF signaling. The different processes discussed in the review either promoted (+) or inhibited (−) by FGF signaling are shown.

The spinal cord cells of vertebrates derive from a region initially specified as neuromesodermal progenitors (NMP) with mixed neural and mesodermal characteristics (Wilson et al., 2009; Henrique et al., 2015; Row et al., 2016), with the exception of those forming the floor plate which in amniotes derive from the node. In chick and mouse, this corresponds to a region of the epiblast adjacent to the early node and the rostral primitive streak. From this population, some cells remain in the ectoderm layer and form most of the spinal cord, while others gastrulate through the primitive streak to become part of the paraxial mesoderm (Wilson et al., 2009; Henrique et al., 2015). Later, with the closure of the caudal neuropore, the NMP region remains in the tailbud from which the caudal spinal cord and mesodermal populations segregate. Overall, this constitutes an ongoing process that takes several days to generate the complete rostrocaudal axis. Different aspects of spinal cord development such as initiation of neurogenesis, ventral patterning and neural crest specification and migration are conditioned by this caudal axis elongation (Figure 1). This is a complex process involving several signaling pathways and gene networks in which the FGF signaling pathway stands as a crucial regulator, maintaining cells in an immature state until they are displaced to a region where they are no longer influenced by it.

FGF signaling acts in numerous stages and tissues during embryonic development and the use of experimental approaches designed to manipulate the FGF signaling pathways at specific stages and tissues has been fundamental to overcome its early roles in implantation, gastrulation, and neural induction. These include treatment of tissue explants with FGF factors or pharmacological antagonists, expression of pathway inhibitors or truncated FGFR proteins that function interfering with the normal function in the neural tube cells *in ovo* and the use of conditional mouse mutants specifically removing FGFs or FGFR in the NMP and its derivatives.

Here, we review the contribution of FGF signaling in the initial process of spinal cord specification and elongation and then we cover the initiation of neurogenesis, ventral patterning, and neural crest specification and migration. We make an effort to identify and separate the different steps in these highly interconnected networks governing spinal cord extension and associated events, focusing on the influence of FGF signaling on the neural tissue. In addition, we have selected some of the evidence supporting the use of FGF to promote regeneration of the lesioned adult spinal cord both acting on spinal cord cells *in vivo* as well as to promote expansion of neural stem cells *in vitro* and their differentiation toward specific neuronal fates for their use for regenerative purposes.

Note: Gene symbols are italicized in all species, but there are specie-specific differences. Thus, gene symbols for human and chick appear all in upper-case; for mouse and rat with only the first letter in upper-case and for fish, gene symbols appear with all letters in lower-case. In the case of protein symbols, they are not italicized and all letters are in upper-case, except in fishes where only the first letter is upper-case (http://www.biosciencewriters.com/Guidelines-for-Formatting-Gene-and-Protein-Names.aspx). When referring to genes from several species they have been separated by a slash.

## FGF signaling pathway: expression of components in the developing spinal cord

Let's first start with a brief introduction of the components of the FGF signaling pathway in the context of spinal cord development. As most signaling pathways, the FGF pathway includes ligands, receptors, modulators, intracellular transducers, and final effectors (Ornitz and Itoh, 2015). The only components exclusive for the pathway are the ligands (up to 23 FGFs have been described in vertebrates) and their receptors of the tyrosin kinase (RTK) type (FGFR1–4 in vertebrates). Other more general players, which are also used by other signaling pathways, such as the pathway inhibitors SPROUTY2, SEF, DUSP6, and the transcription factor effectors of the ETV family, are particularly associated to this pathway as the corresponding mRNAs are highly expressed in regions with high FGF activity and in particular in the caudal NMP region (Chotteau-Lelievre et al., 2001; Karabagli et al., 2002; Corson et al., 2003; Harduf et al., 2005; Lunn et al., 2007). Interestingly, they are themselves downstream targets of the pathway and are thus considered its readouts and have been the basis for the development of pathway activity reporters (Molina et al., 2007; Ekerot et al., 2008). However, as these downstream targets of the pathway are not exclusively activated by the FGF pathway, they do not constitute definitive readouts of the activity of the FGF pathway. The identification of cells where the pathway is truly active is still one of the main difficulties in the analysis of FGF function, as none of the intracellular cascades is specific for FGF signaling and the difference with other RTK pathways may be in the fine tuning of the signaling properties.

**Figure 2:**
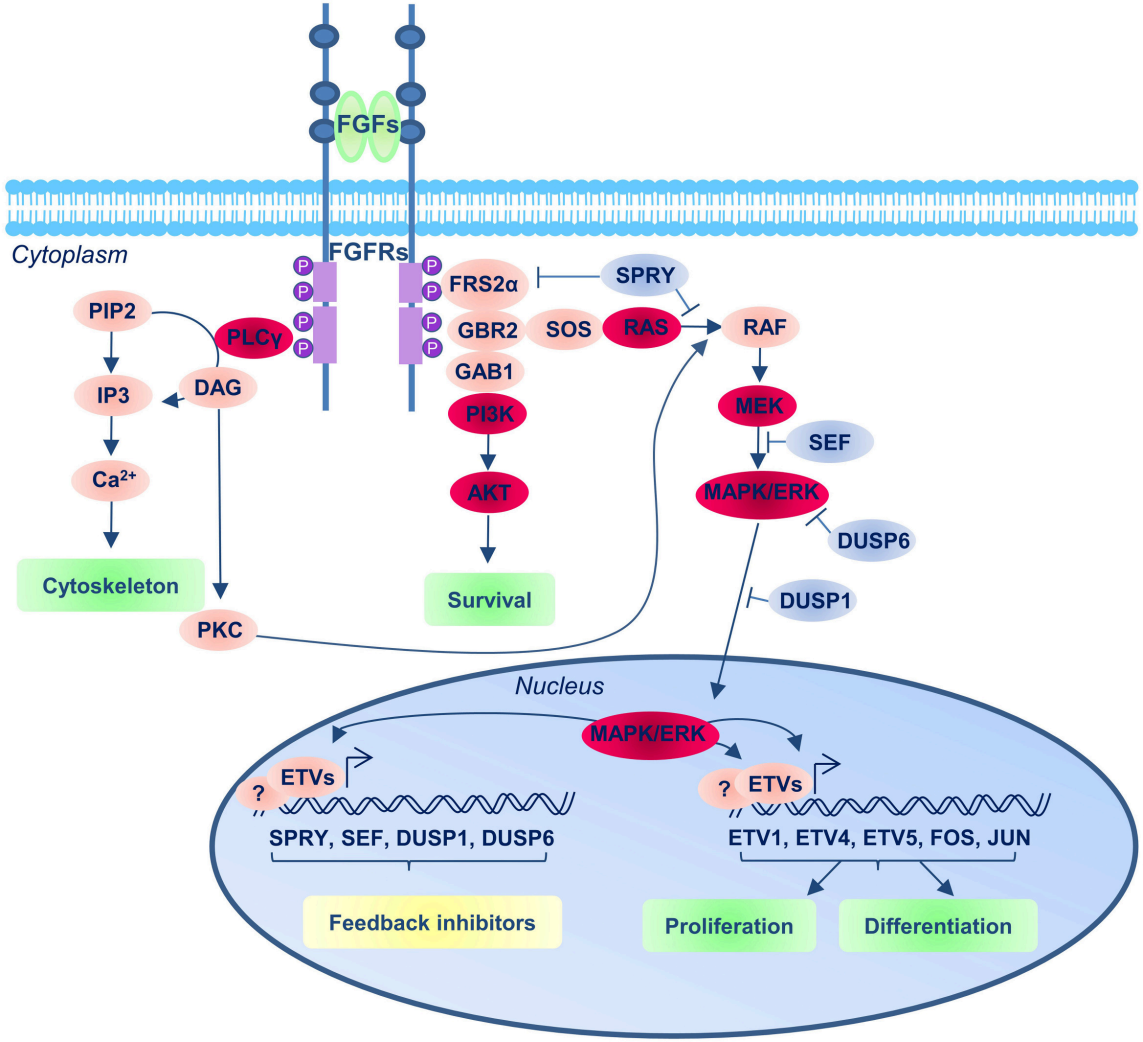
**FGF signaling pathway**. The FGFRs consist of three extracellular immunoglobulin-type domains (D1–D3; blue balls in the receptor), a single-span trans-membrane domain and an intracellular split domain. FGFs interact with the D2 and D3 domains, and promote upon binding receptor dimerization and tyrosine kinase autophosphorylation of the FGFRs that results in the recruitment and assembly of signaling complexes. The main three downstream FGF/FGFR signaling complexes operating in the context of neural development are represented (the red balloons indicate the main components of the pathway): the Ras/MEK/MAPK/ERK; the PI3K/AKT and the PLCγ pathways. The blue balloons indicate the repressor regulators of the pathways.

The three main intracellular cascades that can mediate the FGF signal are: the RAS-MAPK, the PI3K-AKT and the PLPCγ pathways (Figure 2). High levels of MAPK phosphorylation are detected in the NMPs and surrounding area and these depend on the activation of FGF pathway (Lunn et al., 2007). Moreover, most of the effects resulting from FGFR inhibition in this region can also be observed following the inhibition of MEK (MAPK Kinase), suggesting this is the main FGFR downstream pathway in this region (Diez del Corral et al., 2002; Delfino-Machin et al., 2005; Lunn et al., 2007; Martinez-Morales et al., 2011; Olivera-Martinez et al., 2012; Morales et al., 2016).

A gradient of AKT phosphorylation has also been described in the region surrounding the node with higher levels caudally (Dubrulle et al., 2001) but the exposure to PI3K inhibitors does not result in the same effects as blockade of FGFR signaling (Martinez-Morales et al., 2011) and thus the relevance of the AKT pathway in this context has not been addressed further.

**Figure 3:**
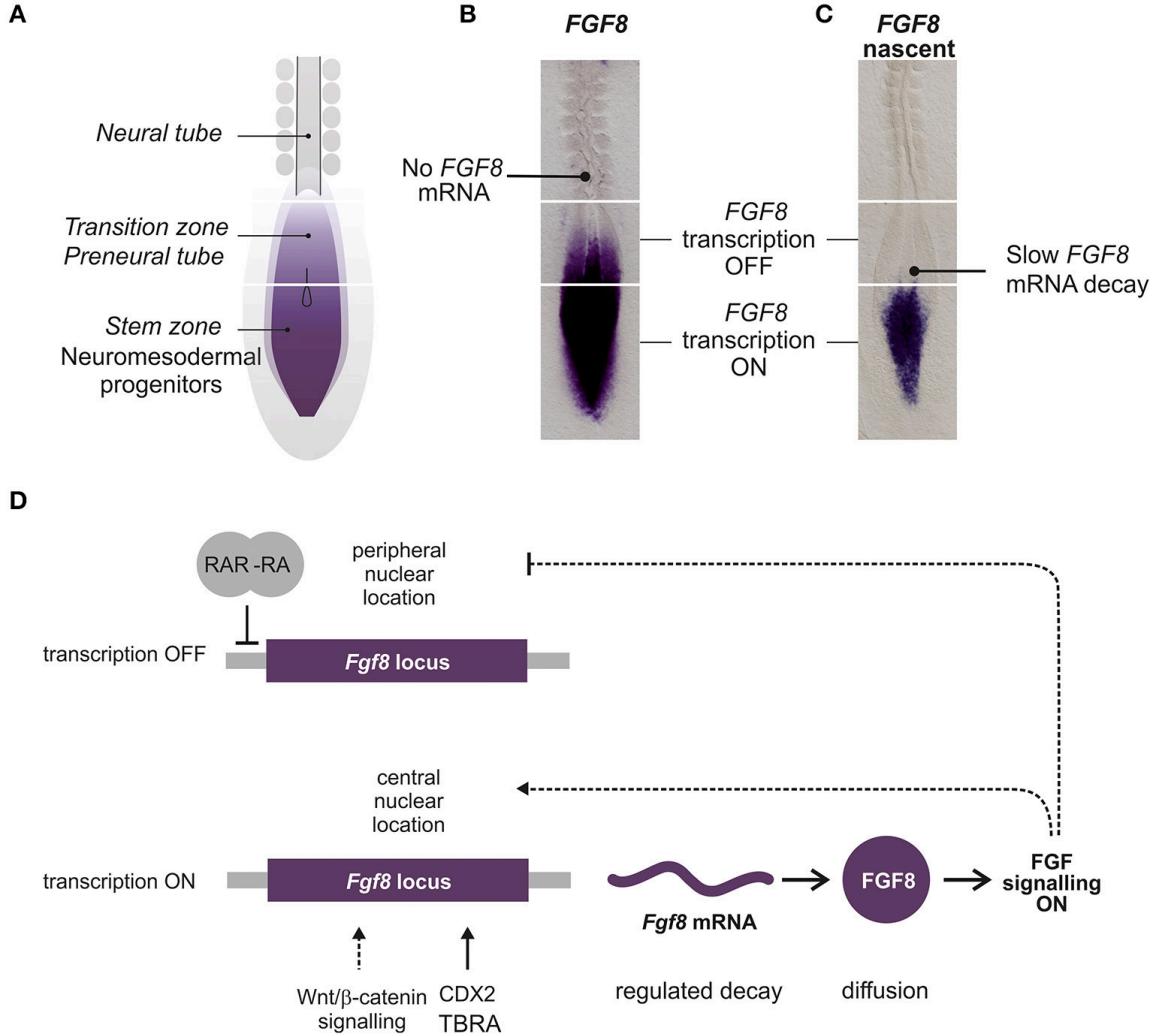
**Regulation of caudal ***FGF8/Fgf8*** expression during spinal cord extension. (A)** Diagram representing the caudal part of a chick embryo where the different regions related to *FGF8* expression are labeled. **(B)** Chick embryo showing *FGF8* mRNA detected by *in situ* hybridization with a full length *FGF8* probe. **(C)** Embryo showing the nascent *FGF8* pre-mRNA detected by *in situ* hybridization with an intronic *FGF8* probe. **(D)** Diagram representing the main factors contributing to the expression of *Fgf8* in the caudal NMP. Lines represent possible direct interactions of transcription factors and dashed lines indirect relations.

The most comprehensive analysis of the expression patterns of FGF signaling related genes in spinal cord development has been performed in the chick. At the stages of chick spinal cord specification, several *FGFs*, including *FGF3, FGF4, FGF8, FGF13, FGF18*, are expressed in the caudal NMP region or surrounding tissues (Karabagli et al., 2002; Delfino-Machin et al., 2005). During later stages (during spinal cord elongation and including tailbud formation) *FGF3, FGF4, FGF13* and *FGF18* become restricted to the primitive streak while *FGF8* is more broadly expressed in the streak, the adjacent NMP region and the ingressing mesoderm (Karabagli et al., 2002; Delfino-Machin et al., 2005). Expression of *FGF8* is highly dynamic as those cells that progress from the NMP state to the spinal cord fate or from the presomitic mesoderm to the somitic mesoderm slowly downregulate their expression (Figures 3A–C). Expression of *FGF4* and *FGF8* in the NMP region (including caudal lateral epiblast and later, the tailbud) continues for several days but declines toward the final stages of somitogenesis and the cessation of axis elongation (Cunningham et al., 2011; Olivera-Martinez et al., 2012).

*FGFR1-3* are initially present in the NMP zone (Karabagli et al., 2002; Lunn et al., 2007; Nishita et al., 2011), but later, only *FGFR1* remains throughout the neural tissue including the NMP region while *FGFR2* is absent there and becomes restricted to the neural tube, rostral to Hensen's node, and *FGFR3* restricts to the neural tube adjacent to somites (Karabagli et al., 2002; Lunn et al., 2007; Nishita et al., 2011).

Similar expression patterns have been described in mouse for those genes analyzed. In mouse, expression of *Fgf3, Fgf4, Fgf8, Fgf17*, and *Fgfr1* has been reported in and around the NMP region (Gofflot et al., 1997; Wahl et al., 2007; Anderson et al., 2016a). In zebrafish, in addition to *fgf4* and *fgf8, fgf17, fgf17b*, and *fgf24* (a zebrafish exclusive gene) have also been shown in or near the tailbud (Reifers et al., 2000; Draper et al., 2003; Cao et al., 2004; Akiyama et al., 2014).

Very little is known about the regulation of expression of FGFRs and FGFs. Most work has been done with *FGF8*, but the control of *FGF8* transcription in the spinal cord NMP region and its progressive downregulation coordinated with embryonic axial extension still constitutes an unsolved enigma (Figure 3). Three regions have been identified with respect to *FGF8* expression: the most caudal region where *FGF8* is actively transcribed (NMP), a more rostral region where transcription is stopped but transcripts remain (known as the transition zone or the preneural tube) and a third most rostral region, adjacent to the mesoderm ready to segment where transcripts are no longer detected (Figures 3A–C).

Several signaling pathways have been shown to influence *Fgf8* expression either promoting or decreasing *Fgf8* levels (Figure 3D). The WNT/β-Catenin pathway is active in the caudal region (Aulehla et al., 2003; Olivera-Martinez and Storey, 2007; Cunningham et al., 2011) and manipulation of the pathway has been shown to affect *Fgf8* expression. Reduced levels of *Fgf8* have been shown in the *Wnt3a* mouse mutant *vestigial tail* (Aulehla et al., 2003) and a further reduction is observed in double *Wnt3a/Wnt8a* mutants (Cunningham et al., 2015b). Furthermore, altering the levels of β-Catenin in the PSM promotes changes in *Fgf8* expression (Aulehla et al., 2008; Dunty et al., 2008). In fact, studies in mouse craniofacial development support a direct role for WNT in *Fgf8* regulation throughout a conserved Tcf/Lef site 2.8 kb upstream of *Fgf8* (Wang et al., 2011). However, no upregulation of *FGF8* by WNT has been observed in chick spinal cord suggesting a more indirect regulation of *FGF8* by the WNT in the context of caudal neural tube (Olivera-Martinez and Storey, 2007). Evidence has also been presented for the requirement of signals from the notochord. In particular, a reduced level of *FGF8* is observed in the absence of the notochord that can be rescued by SHH supplementation (Resende et al., 2010).

An autoregulatory mechanism of FGF activating *FGF8* has been suggested based on the ability of FGF8 to activate the transcriptional repressor *NKX1.2* (previously known as *SAX1;* Bertrand et al., 2000) which when overexpressed can in turn result in increased *FGF8* levels (Sasai et al., 2014). However, exposure of the neural explants or embryos to FGF does not result in activation of *FGF8* expression and inhibition of FGF signaling does not result in decreased *FGF8* or *Fgf8* levels in chick and mouse, respectively (Harrison et al., 2011; Patel et al., 2013), raising the possibility that NKX1.2 may be involved in the stabilization of *FGF8/Fgf8* transcripts.

In addition, although a localized source of a regulator of *FGF8* caudal to Hensen's node has been ruled out (Dubrulle and Pourquie, 2004; Harrison et al., 2011), other caudally active pathways such the HMG-CoA reductase/mevalonate pathway (mediating steroid biogenesis) could be also playing a role in *FGF8* expression activation and maintenance (Olivera-Martinez et al., 2014).

Recent efforts for the characterization of the *Fgf8* gene regulatory region have led to the identification of several regions driving expression around the NMP region (Beermann et al., 2006; Marinic et al., 2013) and furthermore to the identification of CDX2 and TBRA as direct transcriptional activators (Amin et al., 2016). This could explain the decreased *Fgf8* levels that have been observed in *Cdx2* mutants (Savory et al., 2009) but additional activators may also be acting to regulate *Fgf8*, such as WNT/β–Catenin.

In addition to signals maintaining *FGF8*/*Fgf8* expression in the caudal precursor region, progressive downregulation of *FGF8*/*Fgf8* involves cessation of transcription in cells that exit the NMP region. Retinoic acid (RA; which is produced by somites and rostral presomitic mesoderm) has been shown to downregulate *FGF8*/*Fgf8* and reduction in RA signaling (in a vitamin A deprived quail model and in *Raldh2*^−/−^ mutants) results in a rostral expansion of the *FGF8*/*Fgf8* expression domain (Diez del Corral et al., 2003; Molotkova et al., 2005; Vermot and Pourquie, 2005; Sirbu and Duester, 2006; Olivera-Martinez and Storey, 2007; Patel et al., 2013; Kumar and Duester, 2014; Cunningham et al., 2015a). This effect of RA has recently been attributed to direct binding of RA receptor (RAR) to an *RA response-element* (*RARE*) in the regulatory region in the *Fgf8* promoter (Kumar and Duester, 2014; Cunningham and Duester, 2015). This constitutes one of the few examples described where RAR bound to RA would repress gene transcription. Furthermore, additional studies show how NCOR repressors are required for RA repression of *Fgf8* (Kumar et al., 2016).

However, as the forced reduction in RA signaling only promotes a limited expansion of the *FGF8*/*Fgf8* domain, additional mechanisms of transcriptional repression must be involved. One possible theoretical mechanism proposed would involve a caudal diffusing signal transcribed in NMP cells that would repress both its own transcription as well as *FGF8*/*Fgf8* (Harrison et al., 2011). However, to date no such signal has been identified. Moreover, failure of *FGF8*/*Fgf8* to be expressed more anteriorly in spinal cord and somites could be due to a lack of transcriptional activators such as TBRA, CDX, and WNT expression.

Interestingly, the change in the transcriptional state of *Fgf8* locus from active to inactive is associated to a change in its nuclear position from a more central location in the NMP to a more peripheral position in neural tube cells (Patel et al., 2013). This location seems to be regulated by FGF signaling *per se* as inhibiting FGFR signaling results in a more peripheral location of *Fgf8* transcription in the caudal region. However, in spite of the change of location, transcription of *Fgf8* still occurs (Harrison et al., 2011; Patel et al., 2013) suggesting that location of the *Fgf8* locus to the periphery is required but is not sufficient for cessation of expression.

As mentioned above, analysis of active transcription by *in situ* hybridization using intronic *FGF8/Fgf8* probes has shown that the actively transcribing region is rather limited and that cessation of *FGF8/Fgf8* transcription seems to be abrupt (Dubrulle and Pourquie, 2004). However, the high stability of the transcript (which can perdure more than 5 h) is such that a gradient of *FGF8/Fgf8* transcripts can be generated. The mechanism accounting for this high *FGF8/Fgf8* stability however has not been further explored.

Final steps in the regulation of *FGF8*/*Fgf8* expression are the decrease in *FGF8*/*Fgf8* levels associated to the trunk to tail transition and the termination of transcription in the tailbud associated to the termination of axis elongation. Mouse embryos with mutations associated to a prolonged trunk extension *(Gdf11* loss of function mutants or overexpression of OCT4; Aires et al., 2016) show an abnormal increase in *Fgf8* expression in the tailbud region. In the chick, RA derived from the tailbud is also important for the correct termination of *FGF8* expression (Olivera-Martinez et al., 2012) while in the mouse other mechanisms seem to be responsible (Cunningham et al., 2011), as body axis extension continues for a much longer time to form the tail in mouse.

As FGFs are secreted factors, their distribution also depends on their diffusion and transport in the extracellular medium. This has been examined in detail in zebrafish embryos mostly in the context of gastrulation but this may be extended to other situations (reviewed in Bokel and Brand, 2013). There, the binding to heparan sulfates, important constituents of the extracellular matrix, is not only relevant for the activation of the receptor by the ligands but also has an influence on the spread of Fgfs (Yu et al., 2009). The shape of the gradient is also greatly influenced by degradation of Fgf8 that may be largely due to its endocytic removal (Scholpp and Brand, 2004).

Overall, a complex gene regulatory network is in place in the NMP region involving several interconnected signaling pathways that ensures that FGF signaling components are expressed at the appropriate levels for the control of a number of processes that take place as the neural tube extends to form the spinal cord. Given the temporally controlled exposure of cells to FGFs in and around the NMP region, the measurement of the activity of the pathway at the level of the receptor as well as at the different downstream components in fixed tissue and *in vivo*, as the axis extends caudally, would greatly improve our understanding of the coordination of morphogenetic movements and the control of tissue differentiation.

## FGFs and the establishment of the caudal neuromesodermal progenitors and of the spinal cord identity

It has recently become clear that the spinal cord derives progressively from the caudal NMP region which is specified through FGF and WNT actions on sensitized epiblast cells around the primitive streak (Henrique et al., 2015). Specification of the NMP region is initiated at gastrulation stages (Muhr et al., 1999; Delfino-Machin et al., 2005; Nordstrom et al., 2006) within a region around the node and primitive streak characterized by expression of genes such as *NKX1.2, CDX1, CDX2, CDX4*, and *HOXB8* where high MAPK signaling levels are present. This coincidence is maintained during axis extension (Delfino-Machin et al., 2005; Lunn et al., 2007) and reflects the activity of FGF in the control of the expression of those genes (Storey et al., 1998; Muhr et al., 1999; Bel-Vialar et al., 2002; Delfino-Machin et al., 2005; Nordstrom et al., 2006; Sasai et al., 2014). Thus, in the chick embryo, blockade of FGF signaling with a dominant negative form of FGFR (DN-FGFR) and with pharmacological inhibitors results in downregulation of *NKX1.2* and *HOXB8 in vivo* and in explant cultures (Delfino-Machin et al., 2005). In double *Fgf4*; *Fgf8* conditional mutant mice, there is a decrease in *Wnt3a, Wnt5a, Cyp26a1, T-Bra* in the NMP region (Naiche et al., 2011; Boulet and Capecchi, 2012). Similarly, *Fgfr1* conditional mutants also show decreased levels of a number of NMP region genes, such as *Gbx2* and *Cyp26a1* (Wahl et al., 2007). All these results, from the current perspective that stresses the relevance of the NMP cells, suggest that FGF contributes in an important way to the specification of the NMP character in chick and mouse embryos, including genes expressed in NMP and its mesodermal derivatives (*T-BRA*) as well as those expressed in NMP and its neural derivatives (i.e., *NKX1.2*). In the same direction, in Xenopus and zebrafish embryos, expression of a dominant negative form of FGFR/Fgfr (DN-FGFR/DN-Fgfr) results in the loss of markers of NMP and its derivatives (Isaacs et al., 1994; Griffin et al., 1995; Holowacz and Sokol, 1999; Ota et al., 2009).

The NMP give rise to both spinal cord and mesodermal cells during an extended period of time and FGF levels also contribute to preserve the balance between the three cell types. For instance, double *Fgf4*; *Fgf8* conditional mutant mice where defective signaling is restricted to the NMP and its derivatives display dramatic reduction of the presomitic mesoderm markers *Tbx6* (Naiche et al., 2011; Boulet and Capecchi, 2012) and display ectopic neural tubes (Boulet and Capecchi, 2012) similar to the ones observed in *Fgfr* mutant chimeras (Ciruna et al., 1997). In chick, pharmacological inhibition of FGFR results in precocious and caudal expression of the neural tube specific gene *SOX1* (Stavridis et al., 2010). On the other hand, situations with excessive caudal FGF8 signaling such as the *Raldh2* mutant present an imbalanced NMP differentiation favoring mesodermal fate (Cunningham et al., 2015a).

This suggests a requirement of FGF signaling for the promotion of mesodermal or neuromesodermal vs. neural fates (Henrique et al., 2015). Most interestingly, FGF signaling has been shown recently to promote the expression of enzymes that drive the glycolytic metabolic state of the NMP region (Oginuma et al., 2017) that is in turn important for WNT signaling and for restraining the transition from a NMP state to a neural state (Oginuma et al., 2017). Later on, that glycolytic metabolic state in a gradient fashion also operates in presomitic mesoderm development (Bulusu et al., 2017).

In spite of FGF promotion of neuromesodermal and mesodermal fates, FGF signaling in combination with WNT signaling also appears to contribute to the activation and maintenance of the expression of the neural genes *SOX2* and *SOX3* through specific gene regulatory regions (Takemoto et al., 2006; Nishimura et al., 2012) and this might help to prevent the excess production of mesoderm precursors from the NMP (Yoshida et al., 2014).

The precise sequence of exposure of cells to FGF in combination with the other caudal signal WNT as well as the temporal dynamics within the cells may here determine whether cells are maintained in a NMP state, differentiate toward a mesodermal fate or toward a neural fate. This idea has been recently explored with experiments developing *in vitro* methods to generate a population of cells that co-express the NMP genes from mouse and human pluripotent stem cells by timed exposure to FGF2 in combination with WNTs (Gouti et al., 2014; Turner et al., 2014; reviewed in Henrique et al., 2015). This constitutes a good example of how the temporal exposure and competence to interpret FGF signals play an important role in specification of cell fates.

Once spinal cord cells leave the NMP region, FGF is not required for the maintenance of the spinal cord identity. Thus, the spinal cord specific homeobox transcription factor *HOXB8*, that initially requires FGF for its expression in the NMP (Delfino-Machin et al., 2005), remains actively expressed in spinal cord progenitors after the levels of FGF signaling have dropped during axis elongation.

The different mechanisms responsible for the role of FGF in specification of the NMP and then in the balance of mesodermal and neural derivatives may be related to the coactivity with other signals and/or to the temporal sequence of exposure and response of cells to FGF and other signals, as suggested by the cell culture experiments. All these crucial aspects certainly deserve now a thorough analysis within the developing embryo.

## FGFs and the control of spinal cord caudal extension

The most striking feature of embryos where FGF signaling has been diminished (once the early lethality is overcome) is the truncation of the caudal embryonic axis, observed in mouse, Xenopus and zebrafish. Mouse *Fgfr1*^−/−^ embryonic chimeras cannot gastrulate properly and mutant cells tend to accumulate in the tail displaying a short axis (Ciruna et al., 1997; Ciruna and Rossant, 2001). Similarly, *Fgfr1* conditional mutant mice where defective signaling is restricted to the caudal NMP and derivatives (using a *TBra*- driven Cre-line), result in truncated axis at the level of sacral regions (Wahl et al., 2007). An even shorter axis is observed in the double *Fgf4; Fgf8* conditional knock-out (Naiche et al., 2011; Boulet and Capecchi, 2012; either using a TBra- or a Hoxb1-driven Cre-lines). A shortened tail is also apparent in the *Fgf3* null mutant embryos (Anderson et al., 2016a,b). Similarly, in Xenopus and zebrafish the overexpression of DN-FGFR/DN-Fgfr versions also result in truncated embryos (Griffin et al., 1995; Holowacz and Sokol, 1999) and in chick decreased elongation rates have been observed following blockade of FGFR (Benazeraf et al., 2010).

However, in all these situations the lack of FGF signaling affects specification of both mesodermal and neural derivatives and it is therefore not possible to assess whether the defect on elongation is a consequence of an alteration in gastrulation, in the specification of NMP, spinal cord or mesoderm, the result of abnormal motility in the mesoderm (Benazeraf et al., 2010) or whether there is a more specific requirement within the spinal cord population. Support for a more localized role of FGF signaling in spinal cord caudal extension came from analysis of cell distribution after electroporating a *DN-FGFR1* construct in chick NMP region (Mathis et al., 2001; therein referred to as node region). In control experiments, cells could either remain in the NMP region and continue the backward displacement or get incorporated into the neural tube. However, cells with decreased FGF signaling had an increased probability to get incorporated in the neural tube and thus would not be part of the caudally displaced NMP region suggesting some changes in cell adhesion properties of those cells, at least indirectly. In addition, a role of FGF in the maintenance of proliferating cells could also contribute to the extension of the axis (Mathis et al., 2001).

In presomitic mesoderm, axis extension has been shown to involve differential motility of cells along the rostrocaudal axis in a space constrained by lateral boundaries (possibly the lateral plate), with cells moving more in caudal presomitic mesoderm than in the rostral part. Interestingly, in that context, FGF signaling has been shown to promote cell motility (Benazeraf et al., 2010; Lawton et al., 2013). As mentioned before, FGF is required for the transcription of rate limiting enzymes responsible for the glycolytic metabolic state of the NMP that has been shown to be important for cell motility and axis elongation (Oginuma et al., 2017). The mechanism of control of cell motility is still not known but it has been proposed to be related to the ability of localized glycolytic activity to ensure rapid production of ATP for actin polymerization in the forming protrusions of motile cells (Oginuma et al., 2017). In other contexts, FGF has been shown to have chemotaxis properties (Yang et al., 2002) and this has been suggested as an additional mechanism that could in theory contribute to axis extension (Harrison et al., 2011). In any case, given that the spinal cord is composed by epithelial cells and not by mesenchymal cells (as it is the case for presomitic mesoderm) it is unlikely that the same morphogenetic mechanisms are responsible for its extension which may be a more passive process driven by mesoderm.

Recent work on the generation of a population with NMP properties by differentiation of mouse embryonic stem cells (mESCs) in adherent cell culture has shown that these cell aggregates also have the ability to elongate *in vitro* and that this elongation requires FGF signaling, providing an *in vitro* system where this function can be further examined (Turner et al., 2014). In conclusion, there are still many unknowns in relation to the cellular process of spinal cord extension. Most likely, the combination of *in vitro* culture systems together with imaging techniques (both *in vitro* and *in vivo*), the use of biosensors to investigate metabolism in developing embryos (such as the PYRATES mouse line, Bulusu et al., 2017) and *in silico* simulations will greatly contribute to the understanding of the important role of FGF signaling in spinal cord extension.

## FGFs and the control of cell proliferation, cell cycle exit and neuronal differentiation

FGFs play important roles in cell survival and proliferation in many developmental contexts and in particular for neural stem cells and progenitors (Vaccarino et al., 1999; Storm et al., 2006; Maric et al., 2007). In the developing spinal cord, analysis of cell cycle exit (Sechrist and Bronner-Fraser, 1991) and of the early postmitotic marker *NeuroM* (Roztocil et al., 1997) revealed two regions with respect to cell proliferation. *NeuroM*^+^ cells start to appear in the region flanked by somites while no *NeuroM*^+^ cells are found in the more caudal region (the preneural tube) nor in the NMP region, coinciding with the region of influence of FGF signaling (Diez del Corral et al., 2002).

Exposure of the neural tube to FGF at a stage when some cells are already exiting the cell cycle can impair the generation of new *NeuroM* expressing cells (Diez del Corral et al., 2002) and by that way, the onset of neurogenesis. By following the fate of neural progenitors using time lapse imaging, it has been possible to analyze the changes in the dynamics of progenitors associated to FGF exposure (Wilcock et al., 2007). Neural progenitors and stem cells can normally experience three modes of division to give rise to neurons (N) and progenitors and stem cells (P): self-expanding, PP (i.e., giving rise to 2 progenitors or stem cells); self-replacing, PN (i.e., giving rise to a progenitor and a neuron); and self-consuming, NN (i.e., giving rise to 2 neurons). Previous studies in the developing cortex and spinal cord suggest that different modes are associated with different cell cycle duration times, with neuron generating divisions (PN or NN) characterized by a longer cell cycle than PP divisions (Takahashi et al., 1995; Calegari and Huttner, 2003; Calegari et al., 2005; Wilcock et al., 2007).

Upon exposure to FGFs, progenitors only go through PP divisions while no PN nor NN divisions could be observed (Wilcock et al., 2007). These FGF induced PP divisions exhibited the typical short PP cell cycle length while no changes in the range of cleavage plane orientation were observed. Interestingly, a subpopulation of cells was found dividing without contacting the apical membrane and with very short cell cycle times (Wilcock et al., 2007). These data support a role for FGF in the maintenance of cells characterized by a rapid cell cycle that can only generate further progenitors. Interestingly, within the embryo, shorter cell cycle lengths are observed in the region exposed to FGF with respect to the rostral neural tube (Olivera-Martinez et al., 2014) and several cell cycle genes are differentially expressed in the caudal vs. more rostral region and could be regulated by FGF (Lobjois et al., 2004; Olivera-Martinez et al., 2014). One example is *CYCLIN D2*, a cell cycle regulator specifically expressed in the chicken caudal neural plate that can be activated by and requires FGF signaling (Lobjois et al., 2004; Molina and Pituello, in press).

Although exposure to FGF can impede neurogenesis, blockade of FGF signal in explants is not sufficient to drive premature expression of the postmitotic and neurogenesis marker *NeuroM* (Diez del Corral et al., 2002). However, as discussed above, cells subject to interference with FGF signaling in the embryo tend to prematurely leave the NMP region (Mathis et al., 2001) where only proliferating cells are found and it remains to be assessed whether they have alterations in their type of division or cell cycle exit parameters.

A high level of aerobic glycolysis is known to facilitate cancer cell proliferation. Although no significant change in proliferation was observed by Oginuma et al. (2017) in embryos grown in the absence of glucose, more detailed analysis are required in order to determine a possible implication of the FGF dependent changes in metabolism in the control of proliferation during axis extension and more specifically within the spinal cord.

The contribution of FGF to the control of proliferation in the spinal cord discussed above is restricted to cells before or at the onset of neurogenesis and could be equivalent to the ability of FGF2 and FGF8 in the telencephalon to maintain the proliferative symmetrical PP divisions of neuroepithelial cells before the onset of neurogenesis (Raballo et al., 2000; Storm et al., 2006; Maric et al., 2007; Rash et al., 2013). Interestingly, the analysis of the telencephalon of mutant mouse embryos has revealed additional requirements for FGF signaling in proliferation of neurogenic lineages at different steps. At the start of telencephalon neurogenesis, neuroepithelial cells transform into radial glial cells, which divide asymmetrically to generate another radial glia and a postmitotic neuron or a basal progenitor (Gotz and Huttner, 2005) and this transition is promoted by FGF10 (Sahara and O'Leary, 2009). Finally, after neurogenesis has started, it has been demonstrated (using mutants for three FGF receptors) that FGF signaling is required to slow down the progression from radial glia to basal progenitors (Kang et al., 2009; Rash et al., 2011). Similar roles for FGF at later stages of spinal cord development remain to be explored (see below for functions during spinal cord adult neurogenesis).

In addition to a more direct action of FGF on the cell cycle, several FGF dependent pathways could mediate its influence on cell cycle exit and neuronal differentiation before the onset of neurogenesis in the spinal cord. FGF signaling is required for the expression of *DELTA-1*, an important component of the NOTCH signaling pathway involved in mutual inhibition in the NMP region and required to limit precocious cell cycle exit (Akai et al., 2005). Additionally, FGF signaling promotes *WNT8a* expression, which in turn prevents neuronal differentiation (Olivera-Martinez and Storey, 2007).

Manipulation of FGF signaling in chick embryo explants and the use of mouse mutants has shown that FGFs can reduce the levels of RA signaling, a neuronal differentiation promoter (reviewed in Diez del Corral and Morales, 2014) and this would also favor the maintenance of the progenitor state. Double *Fgf4; Fgf8* conditional mutant mouse embryos exhibit increased caudal *RARE-lacZ* reporter expression (Naiche et al., 2011). But, at what level could FGF act on the control of RA signaling? FGF4 and FGF8 can repress the gene encoding the RA-synthesizing enzyme *RALDH2* in the paraxial mesoderm (Diez del Corral et al., 2003). However, the contribution of this repression to the RA levels is probably partial since double *Fgf4; Fgf8* conditional mutant mouse embryos do not exhibit increased *Raldh2* expression (Boulet and Capecchi, 2012). FGF signaling is required for the caudal expression of the RA-degrading enzyme *Cyp26a1* (Wahl et al., 2007) and this could also contribute to the control of RA levels similarly to what has been described in the context of the hindbrain (Gonzalez-Quevedo et al., 2010). FGF4 and FGF8 can also downregulate *RAR*β receptor levels in the spinal cord (Olivera-Martinez and Storey, 2007) and this would affect the sensitivity to RA levels. This receptor gene depends on RA for its activation (Olivera-Martinez and Storey, 2007) and thus its downregulation by FGF could be due to upregulation of *Cyp26a1* but this has not been examined yet.

FGF signaling is also required to prevent precocious activation of *PAX6* and *IRX3* in chick and *Pax6* in mouse (Bertrand et al., 2000; Diez del Corral et al., 2003; Patel et al., 2013), two transcription factors which promote neuronal differentiation (de la Calle-Mustienes et al., 2002; Bel-Vialar et al., 2007). Thus, FGF seems to contribute to a rather complex network that controls proliferation before the onset of neurogenesis maintaining an undifferentiated state. However, open questions still remain: does FGF signaling act differentially on the process of proliferation within NMP and then for promotion of self-renewal of neural progenitors? Does it act differently in the spinal cord than in telencephalon progenitors where it has also been involved in the appearance of intermediate progenitors? What are the cell cycle components modulated by FGF signaling in all these processes?

## FGFs and patterning of spinal cord along the rostro-caudal axis

Once the region of the neural plate giving rise to the spinal cord has been specified (in an FGF dependent way), FGF signaling has an additional role in the further regionalization of the spinal cord along the rostral-caudal axis. The spinal cord presents heterogeneity along the rostro-caudal axis responsible for differences in motor neuron subpopulations, interneuron distribution (Francius et al., 2013; Lai et al., 2016) or neural crest derivatives (Le Douarin et al., 2004). This regionalization, which has been mainly examined in motor neurons, is a consequence of the restricted rostro-caudal expression of *Hox* genes in progenitor cells and subsequently in the resulting postmitotic motor neurons (reviewed in Philippidou and Dasen, 2013).

Experiments in chick embryos have shown that exposure to FGF or electroporation of *FGFs* expressing constructs shifts rostrally the domain of expression of caudal *HOX* mRNAs (*HOXB*6, *HOXC6, HOXB7, HOXB8*, and *HOXA9-B9-C9*) in neural progenitors resulting in an increase in the protein levels of a subset of HOXB proteins (Bel-Vialar et al., 2002; Dasen et al., 2003). FGF signaling appears to act here by activating the transcription factor genes of the Cdx family, known to activate *HOX*/*Hox* gene expression, in particular *cdx2* and *cdx4* in zebrafish (Shimizu et al., 2006), *CDX1* and *CDX2* in the chick (Bel-Vialar et al., 2002), and *Cdx1, Cdx2*, and *Cdx4* in mouse (van den Akker et al., 2002; Amin et al., 2016). Exposure to FGF not only has consequences in the expression of genes in progenitors but also in the resulting motor neurons (Liu et al., 2001; Dasen et al., 2003). Explants of neural tissue fated to give rise to cervical spinal cord do not express HOXC6, HOXC8, HOXC9, or HOXC10 after culture but their exposure to increasing FGF levels results in progressive activation of the production of these proteins suggesting that FGF works in a concentration dependent way. Considering that in the embryo, caudal cells are exposed to FGF for a longer period of time than rostral cells, but not necessarily to higher levels of FGF signaling, this concentration dependent effect has also been interpreted as an effect of the duration of exposure to the FGF morphogen. The mechanism to explain such concentration/time of exposure dependence is still not known but may involve the regulation of genes encoding transcription factors of the CDX family mentioned above.

The role of FGF signaling in this further caudalization, however, has not yet been ascertained by loss of function approaches and therefore, the extent of its contribution to patterning remains an open question. A possible contribution of FGF to rostro-caudal patterning of interneurons has also been suggested (Francius et al., 2013) but has not been explored yet.

## FGFs and ventral pattern (intermediate, ventral and floor plate)

Another regionalization process where FGF signaling plays an essential role is the patterning of the spinal cord along the dorso-ventral (DV) axis which is fundamental for the assignment of neuronal subtype identities such as motor neurons and the different interneuron subtypes (reviewed in Gouti et al., 2015). Specific combinations of transcription factors of the homeodomain and bHLH families are expressed in restricted domains along the DV axis (reviewed in Le Dreau and Marti, 2012). In the ventral/intermediate neural tube this is regulated by the SHH morphogen. The graded distribution of SHH, produced in the ventral midline, results in a graded activation of the pathway and the expression of target genes (reviewed in Briscoe and Small, 2015). In addition, cross-repressive interactions between target genes occur to further delimit and ensure gene expression in the appropriate domains (Briscoe et al., 2000; Kutejova et al., 2016).

**Figure 4:**
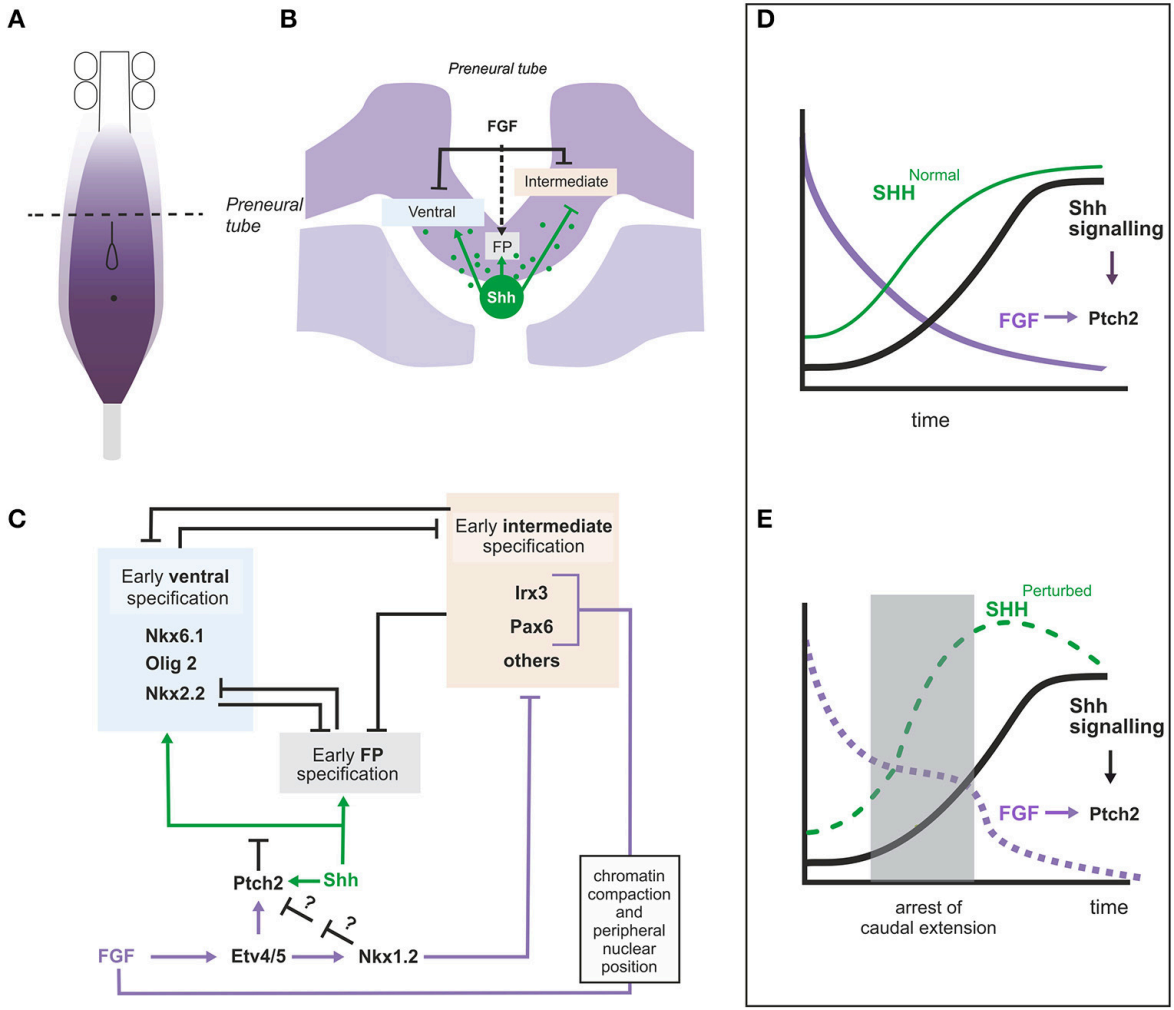
**Role of FGF signaling in the regionalization of the intermediate and ventral neural tube. (A)** Diagram representing the caudal part of an embryo showing the region with active FGF signaling and the rostro-caudal level of the transverse section represented in **(B)**. **(B)** Transverse section at the level of the preneural tube showing the neural tissue and the underlying presomitic mesoderm and notochord. FGF8 (produced by neural and mesoderm tissues) and Shh (produced by notochord) are represented in purple and green respectively and their influence on intermediate, ventral and floorplate specification is shown. The dashed arrow indicates that FGF provides competence for floor plate specification. **(C)** Gene regulatory network relating FGF and Shh signaling in the pre-neural tube where the two signals coincide. Data from chick, mouse or both are included in this figure. **(D,E)** Graphs to illustrate the hypothetical role of the regulation of *Ptch2* by FGF during the initial establishment of the Shh signaling levels. The graphs represent the changing levels of SHH in time at a particular position (within the dorsoventral and rostrocaudal axis). **(D)** In embryos continuously extending their caudal axis, the levels of FGF (purple) at a particular position would decrease constantly while the levels of SHH (green, SHH^Normal^) would increase until they reach their maximum. The levels of Shh signaling thus also increase progressively. **(E)** In embryos where elongation is arrested for some time (shaded area), FGF levels would remain constant during the arrested period, while the levels of SHH (SHH^Perturbed^) would accumulate more rapidly due to the decreased amount of tissue generated through which SHH could diffuse (or be transported). If Shh signaling was dependent exclusively on SHH levels, the level of the signaling would also increase to levels higher than normal and this may result in the irreversible activation of its targets. However, the ability of FGF to activate *Ptch2* and thus downregulate the Shh pathway could serve to limit Shh signaling levels to normal values. FGF signaling levels (decaying in time) are shown in purple.

During spinal cord caudal extension, SHH is expressed in the node and along the derived notochord while in the neural tissue it is expressed in floor plate (FP) cells at the level of the somitic mesoderm. Thus, cells in the preneural tube (the transient spinal cord population derived from NMP and adjacent to presomitic mesoderm) are initially exposed to notochord derived SHH and express some SHH target genes such as *GLI1, PTCH1* and *PTCH2* suggesting that at least low SHH signaling is achieved (Diez del Corral et al., 2003; Morales et al., 2016). However, neural progenitors in the preneural tube do not display expression of the complete repertoire of ventral identity genes, suggesting that the pathway is being modulated in this region. A role for FGF signaling in the control of ventral patterning was first inferred from its ability to repress *PAX6*, a gene expressed in an intermediate domain in the neural tube (Bertrand et al., 2000). Since that observation, a more complex picture has emerged showing that FGF signaling is crucial for controlling the onset of SHH signaling and ventral patterning in the spinal cord (Diez del Corral et al., 2003; Morales et al., 2016) and for the early specification of the most ventral fate, the FP (Sasai et al., 2014; Figure 4).

Forced maintenance of FGF signaling in preneural tube tissue, impairs not only *PAX6* but also other ventral and intermediate patterning genes such as *NKX6.1, NKX6*.2, *IRX3*, and *FOXA2* (Bertrand et al., 2000; Diez del Corral et al., 2003; Novitch et al., 2003). Conversely, interference with FGF signaling in chick embryos results in precocious caudal activation of *PAX6* and *IRX3* (Bertrand et al., 2000; Diez del Corral et al., 2003) and in the dorsal expansion of ventral markers such as OLIG2 and NKX6.1 (Morales et al., 2016). Reduced FGF signaling in mouse embryos results in precocious caudal *Pax6* (Patel et al., 2013) and NKX6.1 expression as well as in alterations in the ventral patterning with an increase in the number of NKX6.1 expressing neural progenitor cells (Morales et al., 2016).

FGF would thus be repressing more or less indirectly the two types of SHH responding genes, ventral genes activated by SHH (*FOXA2* and *NKX6.1*) and intermediate genes repressed by SHH (*PAX6* and *IRX3*) (Briscoe et al., 2000). Repression of *PAX6* and *IRX3* and their mouse homologs seems to involve several mechanisms (Figures 4B,C). FGF signaling promotes chromatin compaction and peripheral nuclear position around the mouse *Pax6* and *Irx3* loci, a chromatin organization associated to transcriptionally inactive loci (Patel et al., 2013). In addition, repression of these genes appears to be mediated by transcriptional repressor NKX1.2, transcriptionally activated by FGF signaling in NMP region (Storey et al., 1998; Bertrand et al., 2000; Sasai et al., 2014; Figure 4C).

A molecular mechanism that accounts for the effect of FGF on genes relying on SHH for their expression has been identified recently (Figure 4C; Morales et al., 2016). FGF can activate the expression of *PTCH2*, one of the SHH receptors that also acts as an inhibitor of the SHH pathway, and can thus restrain expression of SHH targets. Experiments in chick explants have shown that *PTCH2* is expressed in the preneural tube in a SHH and FGF dependent way indicating the existence of an enhanced feedback loop where SHH activates PTCH2 more efficiently in regions of high FGF signaling. This regulation also appears to be conserved in mouse as *Fgfr1* conditional mutant embryos show extremely reduced *Ptch2* levels (Morales et al., 2016). Surprisingly, however, the *Ptch2* gene seems to be largely dispensable as no obvious phenotype has been identified yet in the mouse mutant (Holtz et al., 2013). It is possible that its function is only apparent when the development of the embryo is challenged, for example when the elongation process is altered. If elongation is arrested, the high levels of PTCH2 in the spinal cord precursor may maintain the levels of SHH signaling low and ventral patterning on standby mode until the elongation is restored (Figures 4D,E). In fact, *Ptch2* is required to keep SHH signaling in check in situations of partial deficiency of the other member of the family, *Ptch1*, both in development and in tumorigenesis (Lee et al., 2006; Nieuwenhuis et al., 2006; Holtz et al., 2013; Zhulyn et al., 2015).

In addition, and probably as a result of its role on repressing ventral and intermediate genes, an important role for FGF on the specification of FP cells has been recently identified in chick (Sasai et al., 2014). FP territory, characterized by expression of the ARX1 protein, is induced by the highest levels of SHH that are only achieved in the cells closest to its source and also requires transient FGF exposure (Sasai et al., 2014). Here again, NKX1.2 plays an important role, providing competence to respond to high SHH levels and drive ARX1 expression. Given the repressive interactions between FP specific genes and the ventral and intermediate patterning genes (Cho et al., 2014; Kutejova et al., 2016), one important function for FGF signaling and NKX1.2 here would be to ensure that a region free of expression of non-floor plate factors such as PAX6, IRX3 and NKX2.2 is established in the future FP region (Sasai et al., 2014). Nevertheless, the details of the gene regulatory network are still not elucidated as expression of ARX1 (and other definitive floor plate markers) is only apparent well after the FGF signaling levels have decayed. Here again, the system may be highly redundant as no obvious alterations in the FP have been reported in *Nkx1.2* mutant mice (Simon and Lufkin, 2003), raising the possibility that the related *Nkx1.1* gene could be also playing a role.

Antagonism of the FGF signaling pathway with the RA pathway is also important in the context of ventral patterning as RA is required for expression of several intermediate and ventral genes (chick *NKX6.1, IRX3, PAX6, OLIG2*; Diez del Corral et al., 2003; Novitch et al., 2003; Diez del Corral and Morales, 2014 and mouse *Nkx6.1, Pax6*, and *Olig2*; Molotkova et al., 2005). However, the temporal and quantitative contributions of both FGF and RA pathways in the modulation of ventral specification require a deeper analysis.

## FGFs and neural crest specification

At the most dorsal part of the spinal cord, the development of a specific cell population also requires the participation of FGF signaling: the neural crest cells (NCCs). The neural crest is formed by a transient population of multipotent cells that arise from the dorsal neural tube. Once specified, NCCs undergo a process of epithelium to mesenchyme transition (EMT) that confers NCCs the ability to delaminate and migrate away from the dorsal neural tube, giving rise to NCC derivatives that include craniofacial skeleton, the peripheral nervous system (sensory neurons and glia, sympathetic neurons) and melanocytes, amongst others (Le Douarin and Kalcheim, 1999).

The process of neural crest formation implies the orchestration of a complex gene regulatory network. This involves signaling pathways and transcription factors that are responsible for the sequence of early induction of the NCC during gastrulation; the specification of the neural plate border; the expression of *bona fide* NCC transcription factors and the regulation of numerous downstream effectors involved in EMT, cell adhesion, and cell cycle control, amongst others (Morales et al., 2005; Sauka-Spengler and Bronner-Fraser, 2008). First, parallel to the induction and patterning of the neural plate that generates the central nervous system, at the border between the neural ectoderm and the non-neural ectoderm, the NCCs are specified through a series of steps controlled by FGF, WNT, and BMP signaling pathways (reviewed in Saint-Jeannet and Moody, 2014).

Transient exposure to FGF has been shown to allow neural tube cells to activate NCC markers in response to BMP (Sasai et al., 2014). This seems related to the ability of FGF to repress *PAX6* and *IRX3*, two intermediate neural tube genes which can repress the NCC marker *SNAIL* (Sasai et al., 2014). It has been proposed that the repression of IRX3 and PAX6 by FGF, acting through activation of NKX1.2, is required for the early establishment of a territory competent to NCC specification (see the ventral patterning section for a further discussion on possible mechanisms for FGF regulation of PAX6 and IRX3). However, in FGF deficient conditions impaired NCC specification *in vivo* has not been reported yet. On the contrary, forced reduction of FGF signaling allowed neuroepithelial cells to prematurely initiate the expression of the early NCC specifier *SNAIL2* at caudal levels (Martinez-Morales et al., 2011). This indicates that dorsal neuroepithelial progenitors in the caudal neural tube are maintained in an uncommitted non-NCC state in presence of strong FGF/MAPK signaling pathway (Martinez-Morales et al., 2011). Thus, in the elongating neural tube, as the dorsal neuroepithelial progenitors are progressively exposed to decreasing FGF signaling levels, they initiate the expression of neural crest specifier genes *SNAIL2* and *FOXD3*.

Interestingly, upon reduction of FGF signaling, when those prematurely *SNAIL2* expressing NCCs initiate the expression of other NCCs specifiers such as *FOXD3, SOX5*, and *SOX10* they prematurely start EMT from the neural tube at mid-rostral PSM levels. Essentially, the regulated decrease in FGF signaling is primary responsible for the control of the initiation of NCC specification in the trunk, and as a consequence of that, it controls the timing of EMT and emigration. Subsequent development of trunk NCCs is highly dependent on the development of adjacent somites, which impose a segmented migration and organization to the trunk NCCs and to the derived peripheral nervous system (Sela-Donenfeld and Kalcheim, 1999). Considering that FGF signaling is important both for segmentation of the mesoderm and for the neural crest specification it would constitute an important mechanism of coordination of both tissues.

As it has been described above, FGF and RA signaling can act as opposite gradients, each one negatively regulating the activity of the other. In the context of NCC development, RA signaling produced by the somites does not appear to promote their specification but does trigger the EMT of already specified NCCs (Martinez-Morales et al., 2011). FGF and RA signaling control the timing of EMT and emigration in part through modulation of elements of the BMP and WNT signaling pathways, important signaling cascades operating in the dorsal neural tube (Sela-Donenfeld and Kalcheim, 1999; Burstyn-Cohen et al., 2004). Whereas, RA signaling triggers the initiation of *WNT1* expression in the dorsal neural tube at levels where the NCCs are already specified, FGF signaling prevents the premature expression of *WNT1* (Martinez-Morales et al., 2011).

Moreover, recently it has been established that another FGF ligand, FGF3, coming from the caudal presomitic mesoderm provides another level of regulation of BMP signaling in the spinal cord at tailbud stages. *Fgf3* mutant embryos exhibit axis truncation, increase in neuroepithelial proliferation, delay in neural tube closure and premature neural crest formation (Anderson et al., 2016a). The removal of one copy of NOGGIN, a BMP antagonist, in *Fgf3* mutants, exacerbated all the *Fgf3* phenotypes including premature neural crest specification. Conversely, genetically decreasing BMP signaling in *Fgf3* mutants, via loss of BMP receptor activity, ameliorates morphological defects (Anderson et al., 2016a).

In summary, the data discussed in this section show that there is a limited time window during which the onset of the NCC emigration can be modulated, once those cells have acquired the expression of the essential gene network of the NCC specification program. That window coincides with the region where FGF and RA gradients collide. This FGF function constitutes another example of a general FGF role in controlling the onset of differentiation of cell types as they are generated at the tail end, during trunk axial elongation. Again, the molecular mechanism that allows the cells to interpret and execute that temporal window imposed by FGF signaling is far from understood and remains an important open question within the developmental biology field.

## FGFs and neural stem maintenance in the adult spinal cord

As we have discussed, during the development of the nervous system, the generation of hundreds of subtypes of neurons and glial cells relies upon the relatively fast production, amplification, specification, and differentiation of a pool of neural progenitors and neural stem cells (NSCs). Surprisingly, this strategy is retained to some extent in niches in the adult nervous system throughout lifetime under physiological conditions to generate specific subtypes of neural cells in limited numbers.

Adult NSCs are maintained into adulthood in two main niches, the ventricular-subventricular zone (V-SVZ) adjacent to the lateral ventricles and the subgranular zone (SGZ) in the hippocampus (reviewed in Fuentealba et al., 2012; Christian et al., 2014). Nevertheless, cells with neural stem cell properties can be isolated from most regions of the adult central nervous system, including, for example, the spinal cord (Weiss et al., 1996; Shihabuddin et al., 1997).

In the adult spinal cord, the cells with neural stem cell properties are the ependymal cells (Johansson et al., 1999; Meletis et al., 2008; Barnabe-Heider et al., 2010; Pfenninger et al., 2011). They rarely proliferate under physiological conditions and they mostly give rise to ependymal progeny *in vivo*. It is unclear which signals are responsible for maintaining this population of ependymal cells. However, in other neurogenic niches such as the SGZ of the hippocampus dentate gyrus (DG), the specific deletion of all the FGF receptors that are expressed in DG (*Fgfr1, Fgfr2*, and *Fgfr3*) in adult precursor cells has shown that, FGF signaling is required for neural stem-cell maintenance while an activated FGF receptor expressed in all precursors can increase the number of neurons produced (Kang and Hebert, 2015). The requirement for FGF receptors in maintaining stem but not progenitor cells in the adult hippocampus is reminiscent of their role in maintaining cortical radial glial stem cells during development (Kang et al., 2009).

In spite of the limited expansion of spinal cord ependymal stem cells under normal physiological conditions, their proliferation is dramatically increased after spinal cord injury, giving rise to scar-forming astrocytes as well as to a small population of remyelinating oligodendrocytes (Johansson et al., 1999; Meletis et al., 2008; Barnabe-Heider et al., 2010). More importantly, the ependymal derived astrocytes are essential for repairing the lesions because if their formation is inhibited, the lesions grow deeper over time and a higher number of axonal tracts are lost (Sabelstrom et al., 2013).

The application of FGF2 has been shown to promote functional recovery after spinal cord injury (SCI) in rodents (Lee et al., 1999; Rabchevsky et al., 1999; Yan et al., 2000; Kim et al., 2006). In SCI the recovery is thought to be due to FGF promoting the proliferation of spinal cord neural stem and progenitor cells expressing PAX6, NESTIN, and SOX2 (Shihabuddin et al., 1997; Goldshmit et al., 2014), promoting neuronal survival (Teng et al., 1998, 1999), angiogenesis (Kang et al., 2013), and causing a reduction in injury volume (Lee et al., 1999; Rabchevsky et al., 1999). In addition, FGF2 may reduce glial scar formation and astrogliosis after SCI in the mouse model (Goldshmit et al., 2014). In this situation, FGF2 influences glial cell activation, generating a proregenerative radial/progenitor-like state rather than reactive astrocytes that form scar tissue that are inhibitory to axonal regeneration. It is unclear if these proliferating astrocytes could be derived from the neural stem ependymal cells.

FGF2 also reduces the inflammatory response, as it causes the reduction in macrophage infiltration and cytokine levels (Goldshmit et al., 2014). The reduction in macrophage infiltration may be due to the ability of FGF-2 to reduce the leakiness of the blood-spinal cord barrier after SCI (Kang et al., 2010). Moreover, in combination with transplanting specific cells (Meijs et al., 2004; Kuo et al., 2011; Guzen et al., 2012; Lu et al., 2012) or with special scaffold forming hydrogels FGF1 and FGF2 can provide a proregenerative effect and may have clinical applications in the treatment of SCI (Chen et al., 2015). In fact, FGF1 is currently in clinical trials in human patients with cervical SCI (Wu et al., 2011) and more recently also in combination with special devices and rehabilitation in patients with thoracic SCI (clinical trial, NIH reference NCT02490501).

Since SCI has multiple factors that determine the progress of the injury, a combinatorial therapeutic approach including FGF will most likely be required for the most effective treatment of SCI (reviewed in Siddiqui et al., 2015; Ahuja et al., 2016).

## FGFs promoting neurogenesis in a dish

As a complementary approach and as a way to overcome the limited capacity for self-repair of the mammalian nervous system, efforts are being made to boost the repair process by transplanting exogenous cells into sites of injury (Rosser et al., 2007). FGFs can be used to generate, expand, and differentiate neurons *in vitro* and therefore have a major role to play in such cell replacement therapies.

First, FGF2 together with EGF has been extensively used to promote proliferation and self-renewal of NSCs *in vitro* (Kilpatrick and Bartlett, 1993; Gage et al., 1995; Gritti et al., 1996; Qian et al., 1997; Nelson and Svendsen, 2006). FGF2 converts embryonic stem cells into neural stem cells characterized by rapid self-renewing and the potential to generate neurons, astrocytes, and oligodendrocytes. This acquired tripotent neural stem cell state, which does not exist *in vivo*, provide high proliferative capacity and glial differentiation potential to the treated cells (Palmer et al., 1999; Laywell et al., 2000; Zhang et al., 2001; Gabay et al., 2003; Hack et al., 2004; Pollard et al., 2008). Several studies then showed that FGF2 ventralizes cultured rodent NSCs/NPCs of dorsal origin and induces oligodendrocytes from NSCs derived from regions where oligodendrocytes are not present (Gabay et al., 2003).

FGF2 has also been proved to be involved in neuronal subtype specification, as it has been shown that *in-vitro*-expanded human fetal forebrain-derived NSCs can generate cholinergic neurons with spinal motor neuron properties when treated with FGF2 within a specific time window (Jordan et al., 2009). Moreover, ESC-derived motor neurons, grown using a differentiation program that relies on endogenous embryoid body-derived WNTS, FGFs, and HH signaling, and then grafted isochronically into chick spinal cord, settle in appropriate columnar domains and select axonal trajectories with a fidelity that matches that of their *in vivo* generated counterparts (Peljto et al., 2010). Under those differentiation conditions, it is not clear if increasing FGF levels would increase motor neuron yields without sacrificing the columnar and motor pool subtype diversity achieved.

In the last few years induced pluripotent stem cells (iPSCs) have provided a platform for studying basic human development and disease mechanisms and hold great potential for future cell therapies (Murry and Keller, 2008). Nevertheless, biomedical application of iPSCs depends on the availability of robust cell expansion and differentiation protocols. A recent example is the use of FGFR inhibitor (SU5402) that promoted iPSCs to commit to a NCC cell fate that express specific genes, including *PAX3, SLUG, TFAP-2*α, and *TWIST1* (Jaroonwitchawan et al., 2016).

FGF is also required for the specification of cell types outside the embryonic spinal cord such as the midbrain dopaminergic neurons (Ye et al., 1998). Human pluripotent stem cells have also been successfully converted into dopaminergic neurons using a novel floor plate-based strategy that involves the use of SHH and WNT agonists together with FGF8 and these are efficiently engrafted *in vivo* using rat, mouse and monkey models (Kriks et al., 2011). This could be promising for the development of cell-based therapies in Parkinson's disease.

Finally, it also important to consider the oncogenic risk associated to the mitogenic potential of cells treated with FGFs in transplantation experiments. As a recent example, human cord blood-derived iPSCs have been differentiated into dopaminergic neurons using either FGF2 or BMP/TGF-β inhibitor for neural induction. After transplantation in hemiparkinsonian rats *in vivo*, proliferation still occurred in FGF2-derived grafts (but not in BMP inhibitor treated grafts), resulting in tumor-like growth (Effenberg et al., 2015). Similarly, those effects have also been described for neurospheres derived from hIPSCs and transplanted into spinal cord injured mice (Nori et al., 2015).

## Future directions and challenges

This review highlights the multiple steps in spinal cord development that are regulated by FGF signaling, which may be viewed as a sensor of caudal elongation serving to coordinate different aspects of spinal cord maturation to each other, to adjacent mesoderm and to axial elongation. Further analysis of FGF signaling deficiency in mouse would help ascertain the extent of its contribution to floor plate formation, early neurogenesis, rostro-caudal patterning and neural crest development.

The molecular mechanisms that link FGF signaling specifically to the different functions are still not fully identified but for most of its functions, specific transcriptional targets downstream of the pathway have been proposed. It has been shown that FGF influences transcription by changing the phosphorylation state of transcription factors such as those of the ETV family. The analysis of the regulatory regions of the proposed targets will confirm which of them are more directly regulated. FGF also has an influence on chromatin compaction and nuclear positioning of specific gene loci (Patel et al., 2013) and this may be due, at least in part to the ability of FGF to regulate chromatin modifiers such as histone deacetylase 1 (HDAC1) (Olivera-Martinez et al., 2014).

The detailed regulation of the pathway including the intracellular dynamics of the MAPK pathway with its positive and negative feedbacks (Lake et al., 2016) as well as the involvement of the other FGFR dependent cascades (AKT, PKC) in some of the processes described here also remains largely unexplored. The understanding of the mechanisms responsible for the maintenance of FGF8 and FGF4, the principal ligands in this context, in the NMP and adjacent regions and their progressive downregulation would provide a better insight into axis elongation.

So far, the majority of the literature relies on static views of the expression of ligands and pathway components at different developmental stages. However, it is clear that those are highly dynamic and thus the development of reliable biosensors to measure FGF activity *in vivo* would help to address fundamental questions such as the mechanisms underlying the temporal changes in the response of NMP and its derivatives to FGF.

Throughout the review, we have focused on the similarities that exist in the different vertebrate species but it would also be interesting to understand how FGF functions may have diverged to accommodate the different modes of spinal cord formation (Steventon and Martinez Arias, in press). Equally interesting would be to study the emergence, during chordate evolution, of a function of caudal FGF on development of the caudal neural tube. FGF signaling has been described in the tailbud of the amphioxus cephalochordate embryo and, although only a limited role in somitogenesis has been described (Bertrand et al., 2015), it would be interesting to assess its requirement in spinal cord development.

Some of the functions of FGF described in the development of the spinal cord may also contribute to the maintenance of the ependymal neurogenic niche present in the adult spinal cord and to the functional recovery after SCI shown in rodents and currently under study in humans. Furthermore, the role of FGFs in the maintenance and expansion of neural progenitors as well as their promotion of specific fates *in vitro* supports their therapeutical potential in regenerative biomedicine. The advances in understanding the detailed mechanism underlying FGF function during the development of the central nervous system, and in particular of the spinal cord, should serve to selectively potentiate some of its functions.

## Author contributions

RD and AM jointly conceived, organized and wrote the manuscript.

### Conflict of interest statement

The authors declare that the research was conducted in the absence of any commercial or financial relationships that could be construed as a potential conflict of interest.
